# Supplementary material for: Genetic and Epigenetic Variations of HPV52 in Cervical Precancer
Source: Int J Mol Sci. 2021 Jun 16;22(12):6463. doi: 10.3390/ijms22126463 (PMC8234014; doi:10.3390/ijms22126463)
Supplement: Supplementary file 1 [file ijms-22-06463-s001.zip › ijms-1253058-supplementary.pdf]

Supplementary Data

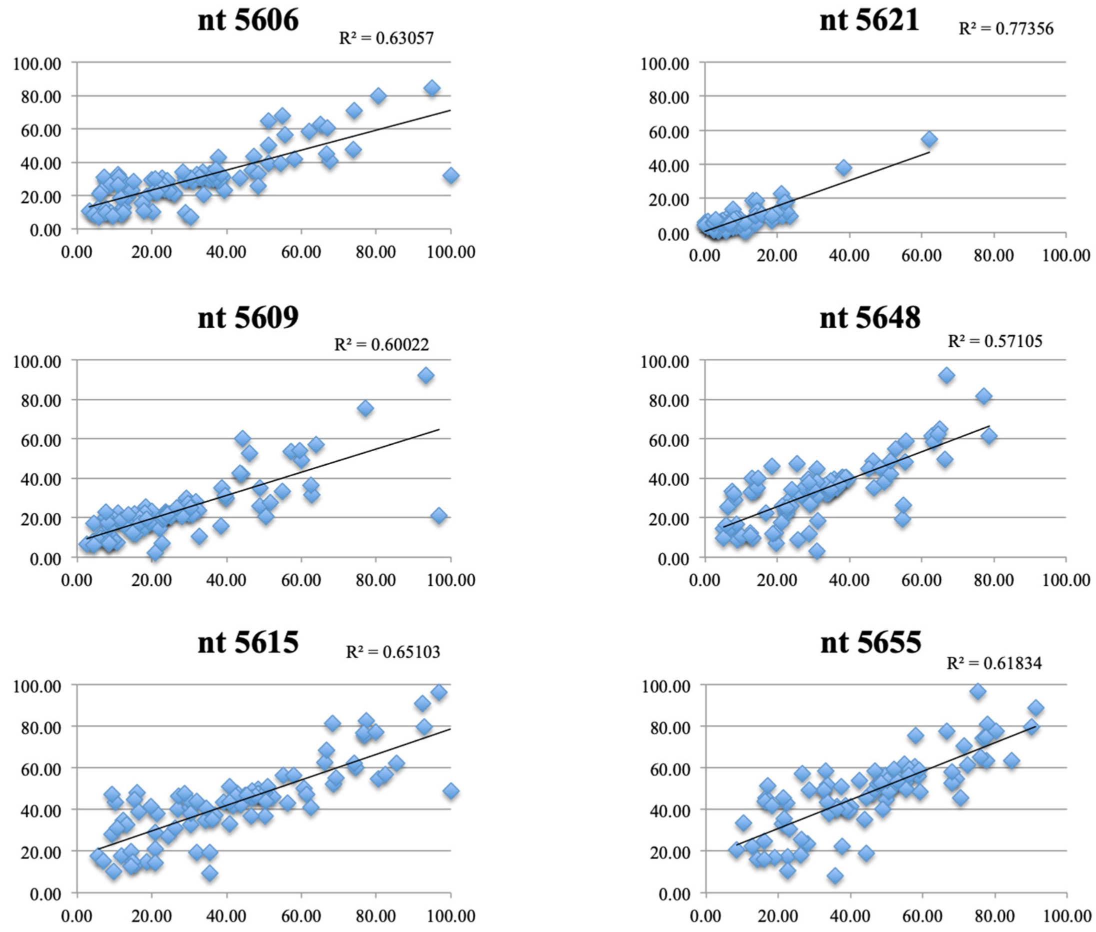

Supplemental Figure S1. Correlation between next-generation sequencing and pyrosequencing for 6 CpG sites within the L1 region. The x-axis shows percentage methylation obtained by pyrosequencing, and the y-axis shows percentage methylation obtained by next-generation sequencing. The figure was created using Microsoft Excel 2008.
